# ﻿Species diversity and taxonomy of *Scytinostroma* sensu stricto (Russulales, Basidiomycota) with descriptions of four new species from China

**DOI:** 10.3897/mycokeys.98.105632

**Published:** 2023-06-13

**Authors:** Yue Li, Wei-Qi Xu, Shi-Liang Liu, Ning Yang, Shuang-Hui He

**Affiliations:** 1 School of Ecology and Nature Conservation, Beijing Forestry University, Beijing 100083, China Beijing Forestry University Beijing China; 2 State Key Laboratory of Mycology, Institute of Microbiology, Chinese Academy of Sciences, Beijing 100101, China State Key Laboratory of Mycology, Institute of Microbiology, Chinese Academy of Sciences Beijing China; 3 Beijing Municipal Research Institute of Eco-Environmental Protection, Beijing 100037, China Beijing Municipal Research Institute of Eco-Environmental Protection Beijing China

**Keywords:** corticioid fungi, Peniophoraceae, phylogeny, white rot, wood-decaying fungi

## Abstract

*Scytinostroma* is species-rich genus in Peniophoraceae, Russulales and has been shown to be polyphyletic. In this study, we performed phylogenetic analyses on the core clade of *Scytinostroma* based on concatenated ITS1-5.8S-ITS2-nrLSU sequence data. Fifteen lineages including four new species from China, *Scytinostromabeijingensis*, *S.boidinii*, *S.subduriusculum*, and *S.subrenisporum*, were recognized. The genus *Michenera* was nested within the *Scytinostroma* s.s. clade in the phylogenetic tree of Peniophoraceae. Sequences of *S.portentosum* (type species) and *S.hemidichophyticum* from Europe formed a strongly supported lineage sister to the *S.portentosum* sample from Canada. It is supposed that the European “*S.portentosum*” is *S.hemidichophyticum*, and the former species is restricted in distribution to North America. *Scytinostromaduriusculum* is supposed to be a species complex. Samples from Sri Lanka (the type locality) formed a lineage sister to those from China, Thailand and Vietnam (described herein as *S.subduriusculum*) and two samples from France that might represent an undescribed species. The four new species are described and illustrated, and an identification key to all the 14 *Scytinostroma* s.s. species worldwide is provided. Until now, seven species of *Scytinostroma* s.s. have been found in China. Our results increased the knowledge of species diversity and taxonomy of corticioid fungi in China.

## ﻿Introduction

The genus *Scytinostroma* Donk sensu lato (Peniophoraceae, Russulales), typified by *S.portentosum* (Berk. & M.A. Curtis) Donk, is characterized by resupinate, effused basidiomes with a smooth to tuberculate hymenophore, a dimitic hyphal system with dextrinoid and cyanophilous skeletal hyphae, presence of gloeocystidia in most species, and subglobose to ellipsoid, variably amyloid or inamyloid, smooth basidiospores (Bernicchia and Gorjón 2010; Liu et al. 2018; Stalpers et al. 2021). It is a widely distributed genus with 42 species level names in Index Fungorum (http://www.indexfungorum.org, accessed on 1 January 2023). Morphologically, *Scytinostroma* can be easily distinguished from other genera of Peniophoraceae by having a dimitic hyphal system and smooth basidiospores. It is similar to *Vararia* P. Karst., which usually differs in having typical dichohyphae (Bernicchia and Gorjón 2010). Liu et al. (2018) demonstrated that *Michenera* Berk. & M.A. Curtis belonged to Peniophoraceae and was closely related to *Scytinostroma*. The two genera are similar in some aspects, such as the texture of basidiome, a dimitic hyphal system, presence of gloeocystidia, but species of *Michenera* have larger basidia and larger, inamyloid, thick-walled basidiospores. Based on morphology, Stalpers et al. (2021) transferred the two species of *Michenera* to *Scytinostroma* and treated the former genus as a synonym of the latter.

Larsson and Larsson (2003) and Miller et al. (2006) showed that five species of *Scytinostroma*, *S.galactinum* (Fr.) Donk, *S.jacksonii* Boidin, *S.ochroleucum* Donk, *S.odoratum* (Fr.) Donk and *S.portentosum* (type species), occurred on five distinct branches that are distantly separated in Peniophoraceae. Leal-Dutra et al. (2018) built the genus *Baltazaria* Leal-Dutra, Dentinger & G.W. Griff. for *S.galactinum* and other three species, *S.neogalactinum* Boidin & Lanq., *S.eurasiaticogalactinum* Boidin & Lanq. and *Parapteruliciumoctopodites* Corner. Our preliminary phylogenetic analyses showed that some specimens recently collected from China clustered with *S.portentosum* (type species) and several other species, which represented the core clade of *Scytinostroma*. In order to understand the species diversity within this clade, we carried out phylogenetic analyses of Peniophoraceae based on concatenated ITS1-5.8S-ITS2-nrLSU sequence data, focusing on samples of *Scytinostroma* s.s. worldwide. Fifteen species-level lineages were recognized in the phylogenetic tree. Among them, four lineages are new and here described and illustrated as *S.beijingensis*, *S.boidinii*, *S.subduriusculum*, and *S.subrenisporum* spp. nov.

## ﻿Materials and methods

### ﻿Specimen collection

In situ photos of specimens were taken with a Canon camera EOS 70D (Canon Corporation, Japan). Specimens were dried with a portable dryer, labelled, and then stored in a freezer at minus 40 °C for two weeks to kill the insects and their eggs before proceeding with morphological and molecular studies. Voucher specimens are deposited at the herbarium of Beijing Forestry University, Beijing, China (BJFC).

### ﻿Morphological studies

Thin, freehand sections were made from dried basidiomes and mounted in 2% (weight/volume) aqueous potassium hydroxide (KOH) and 1% (w/v) aqueous phloxine. Amyloidity and dextrinoidity of hyphae and basidiospores were checked in Melzer’s reagent (IKI). Cyanophily of hyphal and basidiospore walls were observed in 1% (w/v) cotton blue in 60% (w/v) lactic acid (CB). Microscopic examinations were carried out with a Nikon Eclipse 80i microscope (Nikon Corporation, Japan) at magnifications up to 1000×. Drawings were made with the aid of a drawing tube. The following abbreviations are used: IKI– = neither amyloid nor dextrinoid, CB+ = cyanophilous, CB– = acyanophilous, SA+ = positive reaction in Sulphobenzaldehyde, SA– = negative reaction in Sulphobenzaldehyde, L = mean spore length, W = mean spore width, Q = L/W ratio, n (a/b) = number of spores (a) measured from the number of specimens (b). Color codes and names follow Kornerup and Wanscher (1978).

### ﻿DNA extraction and sequencing

A CTAB plant genomic DNA extraction kit, DN14 (Aidlab Biotechnologies Co., Ltd., Beijing, China) was used to extract total genomic DNA from dried specimens, then amplified by the polymerase chain reaction (PCR), according to the manufacturer’s instructions. The ITS1-5.8S-ITS2 region was amplified with the primer pair ITS5/ITS4 (White et al. 1990) using the following protocol: initial denaturation at 95 °C for 4 min, followed by 34 cycles at 94 °C for 40 s, 58 °C for 45 s and 72 °C for 1 min, and final extension at 72 °C for 10 min. The D1-D2 region of the nucleic ribosomal LSU was amplified with the primer pair LR0R/LR7 (https://sites.duke.edu/vilgalyslab/rdna_primers_for_fungi/) employing the following procedure: initial denaturation at 94 °C for 1 min, followed by 34 cycles at 94 °C for 30 s, 50 °C for 1 min and 72 °C for 1.5 min, and final extension at 72 °C for 10 min. DNA sequencing was performed at Beijing Genomics Institute, and newly generated sequences were deposited in GenBank (https://www.ncbi.nlm.nih.gov/). BioEdit v.7.0.5.3 (Hall 1999) and Geneious Basic v.11.1.15 (Kearse et al. 2012) were used to review the chromatograms and for contig assembly.

### ﻿Phylogenetic analyses

The dataset of concatenated ITS1-5.8S-ITS2-nrLSU sequences of the Peniophoraceae was analyzed. *Amylostereumchailletii* (Pers.) Boidin and *A.laevigatum* (Fr.) Boidin were selected as the outgroup (Larsson and Larsson 2003; Xu et al. 2023). Sequences including those from Larsson and Larsson (2003) were partitioned to ITS1, 5.8S, ITS2 and nrLSU, and then aligned separately using MAFFT v.74 (http://mafft.cbrc.jp/alignment/server/, Katoh et al. 2017) with the G-INS-I iterative refinement algorithm and optimized manually in BioEdit v.7.0.5.3. The separate alignments were then concatenated using Mesquite v.3.5.1 (Maddison and Maddison 2018). The dataset was deposited in TreeBase (http://treebase.org/treebase-web/home.html, submission ID: 30453).

Maximum likelihood (ML) analyses, and Bayesian inference (BI) were carried out by using RAxML v.8.2.10 (Stamatakis 2014) and MrBayes 3.2.6 (Ronquist et al. 2012), respectively. In ML analysis, statistical support values were obtained using rapid bootstrapping with 1000 replicates, with default settings for other parameters. For BI, the best-fit substitution model was estimated with jModeltest v.2.17 (Darriba et al. 2012). Four Markov chains were run for 2,000,000 generations for the dataset; until the split deviation frequency value was lower than 0.01. Trees were sampled every 100^th^ generation. The first quarter of the trees, which represented the burn-in phase of the analyses, were discarded, and the remaining trees were used to calculate posterior probabilities (BPP) in the majority rule consensus tree.

## ﻿Results

### ﻿Phylogenetic analyses

**Figure 1.**
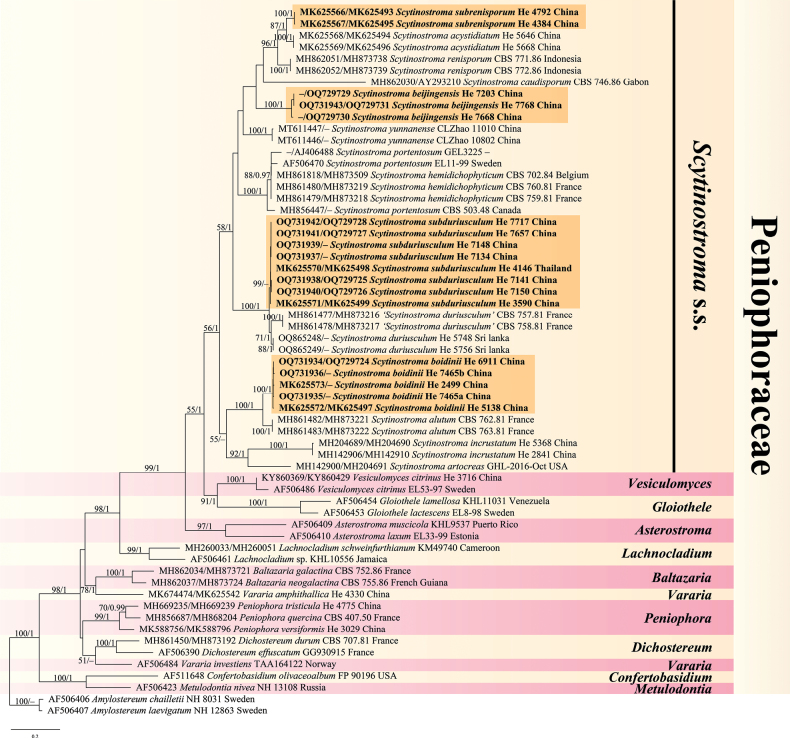
Phylogenetic tree of ML analysis from the ITS1-5.8S-ITS2-nrLSU sequences of Peniophoraceae taxa. Branches are labelled with likelihood bootstrap values (≥ 50%, first) and Bayesian posterior probabilities (≥ 0.95, second). New species are set in bold and highlighted.

The concatenated ITS1-5.8S-ITS2-nrLSU dataset contained 58 ITS and 52 nrLSU sequences from 61 samples, representing 33 ingroup taxa and the outgroup (Table 1), and had an aligned length of 2628 characters. jModelTest suggested GTR+I+G, K80+I, HKY+I+G, GTR+I+G to be the best-fit models of nucleotide evolution for ITS1, 5.8S, ITS2, and nrLSU markers, respectively, for the Bayesian analysis. The average standard deviation of split frequencies of BI was 0.005711 at the end of the run. BI analyses resulted in almost identical tree topologies with the ML analysis. Only the ML tree is provided in Fig. 1 with the likelihood bootstrap values (≥ 50%, first) and Bayesian posterior probabilities (≥ 0.95, second) labelled along the branches.

**Table 1.** Species and sequences used in the phylogenetic analyses. New species are set in bold with type specimens indicated with an asterisk (*).

Species	Specimen No.	Locality	GenBank Accession No.		Reference
			ITS	nrLSU	
*Asterostromalaxum*	EL33-99	Estonia	AF506410	AF506410	Larsson and Larsson 2003
*Asterostromamuscicola*	KHL9537	Puerto Rico	AF506409	AF506409	Larsson and Larsson 2003
*Baltazariagalactina*	CBS 752.86	France	MH862034	MH873721	Vu et al. 2019
*Baltazarianeogalactina*	CBS 755.86	French Guiana	MH862037	MH873724	Vu et al. 2019
*Confertobasidiumolivaceoalbum*	FP 90196	USA	AF511648	AF511648	Larsson and Larsson 2003
*Dichostereumdurum*	CBS 707.81	France	MH861450	MH873192	Vu et al. 2019
*Dichostereumeffuscatum*	GG930915	France	AF506390	AF506390	Larsson and Larsson 2003
*Gloiothelelactescens*	EL8-98	Sweden	AF506453	AF506453	Larsson and Larsson 2003
*Gloiothelelamellosa*	KHL11031	Venezuela	AF506454	AF506454	Larsson and Larsson 2003
*Lachnocladiumschweinfurthianum*	KM49740	Cameroon	MH260033	MH260051	Leal-Dutra et al. 2018
*Lachnocladium* sp.	KHL10556	Jamaica	AF506461	AF506461	Larsson and Larsson 2003
*Metulodontianivea*	NH 13108	Russia	AF506423	AF506423	Larsson and Larsson 2003
*Peniophoraquercina*	CBS 407.50	France	MH856687	MH868204	Vu et al. 2019
*Peniophoratristicula*	He 4775	China	MH669235	MH669239	Liu and He 2018
*Peniophoraversiformis*	He 3029	China	MK588756	MK588796	Xu et al. 2023
*Scytinostromaacystidiatum*	He 5646	China	MK625568	MK625494	Present study
*Scytinostromaacystidiatum*	He 5668	China	MK625569	MK625496	Present study
*Scytinostromaalutum*	CBS 762.81	France	MH861482	MH873221	Vu et al. 2019
*Scytinostromaalutum*	CBS 763.81	France	MH861483	MH873222	Vu et al. 2019
*Scytinostromaartocreas*	GHL-2016-Oct	USA	MH142900	MH204691	Liu et al. 2018
***Scytinostromabeijingensis***	**He 7203**	**China**	–	**OQ729729**	**Present study**
***Scytinostromabeijingensis***	**He 7668**	**China**	–	**OQ729730**	**Present study**
***Scytinostromabeijingensis***	He 7768*	**China**	**OQ731943**	**OQ729731**	**Present study**
***Scytinostromaboidinii***	**He 2499**	**China**	**MK625573**	–	**Present study**
***Scytinostromaboidinii***	**He 5138**	**China**	**MK625572**	**MK625497**	**Present study**
***Scytinostromaboidinii***	He 6911*	**China**	**OQ731934**	**OQ729724**	**Present study**
***Scytinostromaboidinii***	**He 7465a**	**China**	**OQ731935**	–	**Present study**
***Scytinostromaboidinii***	**He 7465b**	**China**	**OQ731936**	–	**Present study**
*Scytinostromacaudisporum*	CBS 746.86	Gabon	MH862030	AY293210	Vu et al. 2019; Binder et al. 2005
*Scytinostromaduriusculum*	He 5748	Sri Lanka	OQ865248	–	Present study
*Scytinostromaduriusculum*	He 5756	Sri Lanka	OQ865249	–	Present study
‘*Scytinostromaduriusculum*’	CBS 757.81	France	MH861477	MH873216	Vu et al. 2019
‘*Scytinostromaduriusculum*’	CBS 758.81	France	MH861478	MH873217	Vu et al. 2019
*Scytinostromahemidichophyticum*	CBS 702.84	Belgium	MH861818	MH873509	Vu et al. 2019
*Scytinostromahemidichophyticum*	CBS 759.81	France	MH861479	MH873218	Vu et al. 2019
*Scytinostromahemidichophyticum*	CBS 760.81	France	MH861480	MH873219	Vu et al. 2019
*Scytinostromaincrustatum*	He 2841	China	MH142906	MH142910	Liu et al. 2018
*Scytinostromaincrustatum*	He 5368	China	MH204689	MH204690	Liu et al. 2018
*Scytinostromaportentosum*	CBS 503.48	Canada	MH856447	AF518723	Vu et al. 2019
*Scytinostromaportentosum*	EL11-99	Sweden	AF506470	AF506470	Larsson and Larsson 2003
*Scytinostromaportentosum*	GEL3225	–	–	AJ406488	Langer 2002
*Scytinostromarenisporum*	CBS 771.86	Indonesia	MH862051	MH873738	Vu et al. 2019
*Scytinostromarenisporum*	CBS 772.86	Indonesia	MH862052	MH873739	Vu et al. 2019
***Scytinostromasubduriusculum***	**He 3590**	**China**	**MK625571**	**MK625499**	**Present study**
***Scytinostromasubduriusculum***	**He 4146**	**Thailand**	**MK625570**	**MK625498**	**Present study**
***Scytinostromasubduriusculum***	**He 7134**	**China**	**OQ731937**	–	**Present study**
***Scytinostromasubduriusculum***	**He 7141**	**China**	**OQ731938**	**OQ729725**	**Present study**
***Scytinostromasubduriusculum***	**He 7148**	**China**	**OQ731939**	–	**Present study**
***Scytinostromasubduriusculum***	**He 7150**	**China**	**OQ731940**	**OQ729726**	**Present study**
***Scytinostromasubduriusculum***	He 7657*	**China**	**OQ731941**	**OQ729727**	**Present study**
***Scytinostromasubduriusculum***	**He 7717**	**China**	**OQ731942**	**OQ729728**	**Present study**
***Scytinostromasubrenisporum***	**He 4384**	**China**	**MK625567**	**MK625495**	**Present study**
***Scytinostromasubrenisporum***	He 4792*	**China**	**MK625566**	**MK625493**	**Present study**
*Scytinostromayunnanense*	CLZhao 10802	China	MT611446	–	Wang et al. 2020
*Scytinostromayunnanense*	CLZhao 11010	China	MT611447	–	Wang et al. 2020
*Varariaamphithallica*	He 4330	China	MK674474	MK625542	Present study
*Varariainvestiens*	TAA164122	Norway	AF506484	AF506484	Larsson and Larsson 2003
*Vesiculomycescitrinus*	He 3716	China	KY860369	KY860429	Present study
*Vesiculomycescitrinus*	EL53-97	Sweden	AF506486	AF506486	Larsson and Larsson 2003
**OUTGROUP**					
*Amylostereumchailletii*	NH 8031	Sweden	AF506406	AF506406	Larsson and Larsson 2003
*Amylostereumlaevigatum*	NH 12863	Sweden	AF506407	AF506407	Larsson and Larsson 2003

In the tree, *Scytinostroma* s.s. clade received a moderately strong support value in ML analysis (bootstrap value = 56) but a strong value in BI (Bayesian posterior probabilities = 1). Four new distinct lineages corresponding to *Scytinostromabeijingensis*, *S.boidinii*, *S.subduriusculum* and *S.subrenisporum* spp. nov. were recognized. Sequences of *S.portentosum* and *S.hemidichophyticum* from Europe formed a strongly supported lineage sister to the *S.portentosum* sample from Canada. Samples of *S.duriusculum* from France and those from Sri Lanka (the type locality) formed a lineage sister to *S.subduriusculum*. *Scytinostromaincrustatum* (S.H. He, S.L. Liu & Nakasone) K.H. Larss. and *S.artocreas* (Berk. & M.A. Curtis) K.H. Larss.,which were formerly placed in *Michenera*, were nested within *Scytinostroma* s.s. clade.

### ﻿Taxonomy

#### ﻿ Scytinostroma beijingensis

Taxon classificationFungiRussulalesPeniophoraceae

Yue Li, S.L. Liu & S.H. He sp. nov.

7D87E85A-A623-5E9C-97B8-B15CE2880D7B

 848268

Figs 2 , 3

##### Type.

China, Beijing, Haidian District, Yangtaishan Forest Park, on dead *Pyrus* tree, 4 September 2022, He 7768 (BJFC 038905, holotype).

**Figure 2.**
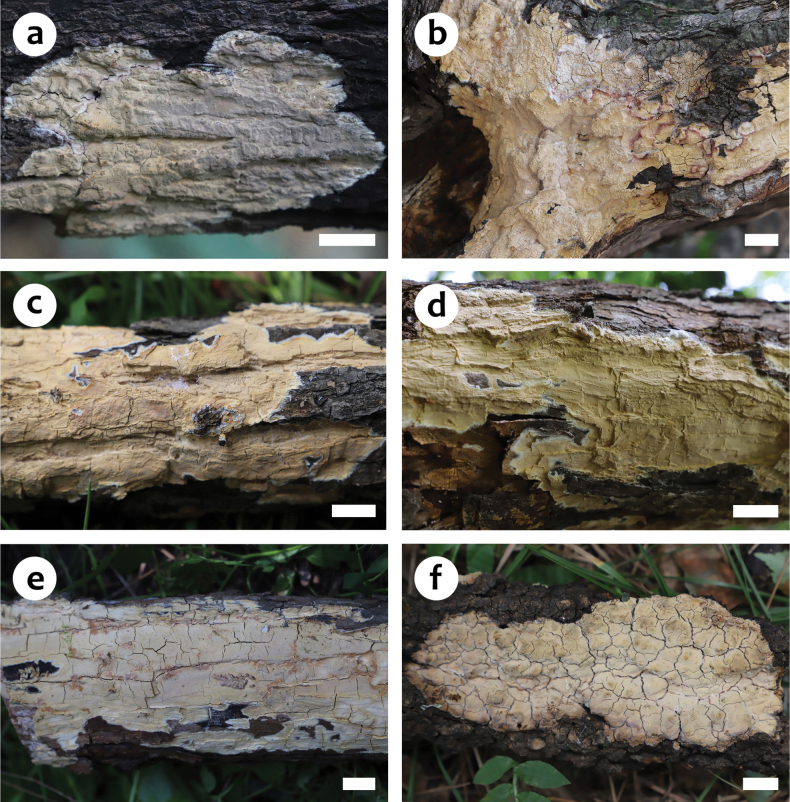
Basidiomes of *Scytinostromabeijingensis***a** He 7768 (BJFC 038905, holotype) **b** He 7201 (BJFC 036518) **c** He 7203 (BJFC 036520) **d** He 7220 (BJFC 036537) **e** He 7668 (BJFC 038804) **f** He 7759 (BJFC 038896). Scale bars: 1 cm (**a–f**).

**Figure 3.**
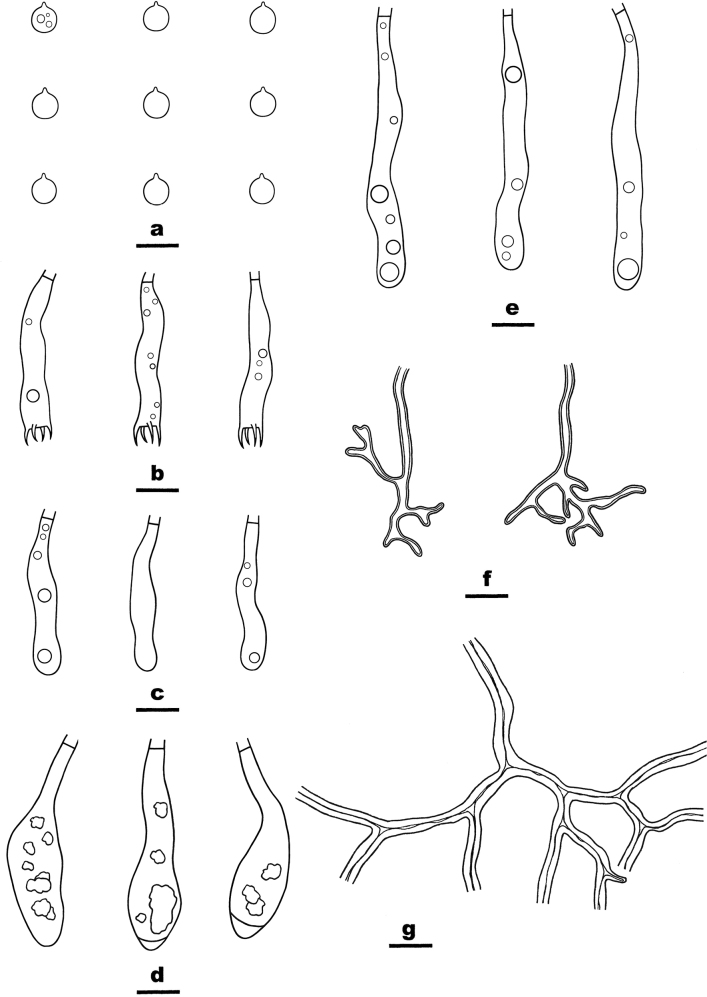
Microscopic structures of *Scytinostromabeijingensis* (from the holotype He 7768) **a** basidiospores **b** basidia **c** basidioles **d**, **e** gloeocystidia **f** skeletal hyphae from hymenium **g** skeletal hyphae from subiculum. Scale bars: 10 µm (**a–g**).

##### Etymology.

Refers to the type locality in Beijing, China.

##### Fruiting body.

Basidiomes annual, resupinate, widely effused, closely adnate, inseparable from substrate, coriaceous, first as small patches, later confluent up to 12 cm long, 4.5 cm wide, up to 200 µm thick in section. Hymenophore smooth, greyish yellow (4B5) to greyish orange (5B5), unchanged in KOH, not cracked or deeply cracked with age; margin thinning out, adnate, fimbriate, white or concolorous with hymenophore surface. Context yellow.

##### Microscopic structures.

Hyphal system dimitic. Context thickening, compact. Generative hyphae rare, scattered, simple-septate, colorless, thin-walled, 2–3 µm in diam., IKI–, CB–. Skeletal hyphae dominant, colorless to yellow, distinctly thick-walled, moderately branched, 2.5–4 µm in diam., weakly dextriniod, CB+. Catahymenium composed of skeletal hyphae, gloeocystidia, basidia and basidioles. Skeletal hyphae abundant, similar to those in the context, but strongly dextrinoid, frequently dichotomous-branched with acute tips, 1–2 µm wide at lowest part. Gloeocystidia abundant, SA+, with two shapes (1) ventricose, colorless, thin- to slightly thick-walled, mostly embedded, usually with contents, 28–40 × 8–15 µm; (2) subcylindrical, colorless, thin- to slightly thick-walled, mostly projecting beyond the hymenium, usually with contents, 45–65 × 5–7 µm. Basidia subcylindrical, slightly curved, thin-walled, colorless, smooth, with four sterigmata and a basal simple septum, 30–36 × 4.5–6.5 µm; basidioles in shape similar to basidia, but slightly smaller. Basidiospores subglobose, with a distinct apiculus, thin-walled, colorless, smooth, occasionally with oil-drops, amyloid, CB–, 5.5–6.5 (–6.8) × (5–) 5.2–6.2 (–6.5) µm, L = 5.9 µm, W = 5.8 µm, Q = 1.01–1.02 (n = 90/3).

##### Additional specimens examined.

China, Beijing, Haidian District, Jiufeng Forest Park, on dead *Pyrus* tree, 26 August 2022, He 7759 (BJFC 038896); Xiangshan Park, on dead *Pyrus* tree, 16 July 2022, He 7668 (BJFC 038804); Miyun District, Yunmengshan Scenic Spot, on dead *Pyrus* branch, 7 August 2021, He 7201 (BJFC 036518) & He 7203 (BJFC 036520) & He 7220 (BJFC 036537).

##### Notes.

*Scytinostromabeijingensis* is characterized by having two kinds of gloeocystidia and short branched skeletal hyphae in hymenium, and growing on *Pyrus*. In the phylogenetic tree (Fig. 1), *S.beijingensis* formed a distinct lineage with strong support values that is sister to the clade comprising *S.renisporum* Boidin, Lanq. & Gilles, *S.subrenisporum*, *S.acystidiatum* Q.Y. Zhang, L.S. Bian & Q. Chen and *S.caudisporum* Boidin, Lanq. & Gilles. *Scytinostromarenisporum* differs from *S.beijingensis* by having cylindrical, subclavate or fusoid gloeocystidia (20–35 × 6–10 µm), ovoid to reniform basidiospores (5.2–6.5 × 3.2–4.8 µm) and a distribution in Côte d’Ivoire, western Africa (Boidin and Lanquetin 1987). *Scytinostromasubrenisporum* and *S.acystidiatum* can be easily distinguished from *S.beijingensis* by the absence of gloeocystidia (Zhang et al. 2023). *Scytinostromacaudisporum* is unique in the group for its distinctly large basidiospores (15–30 × 3–3.5 µm, Boidin and Lanquetin 1987).

#### ﻿ Scytinostroma boidinii

Taxon classificationFungiRussulalesPeniophoraceae

Yue Li, S.L. Liu & S.H. He sp. nov.

AF3960CF-734C-5E44-A411-1F19AC3DD68C

 848267

Figs 4 , 5

##### Type.

China, Beijing, Mentougou District, Xiaolongmen Forest Park, on dead angiosperm branch, 28 August 2020, He 6911 (BJFC 033860, holotype).

**Figure 4.**
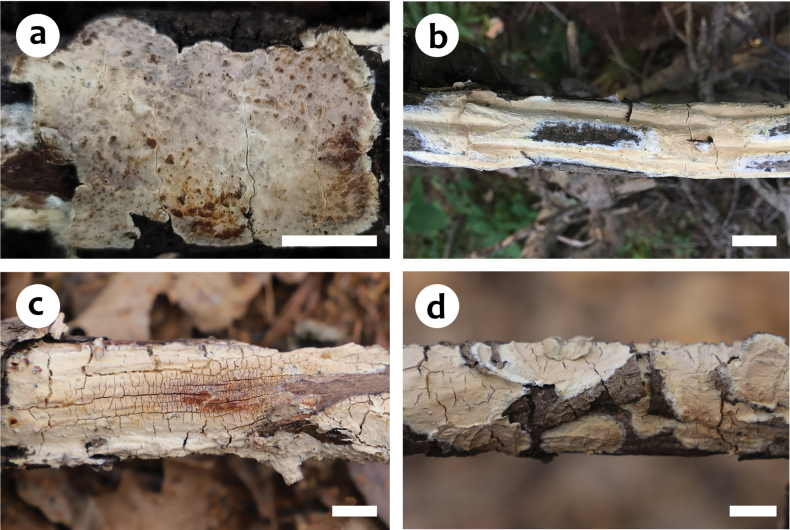
Basidiomes of *Scytinostromaboidinii***a** He 6911 (BJFC 033860, holotype) **b** He 4985 (BJFC 024503) **c** He 7465a (BJFC 038600) **d** He 7465b (BJFC 038601). Scale bars: 1 cm (**a–d**).

**Figure 5.**
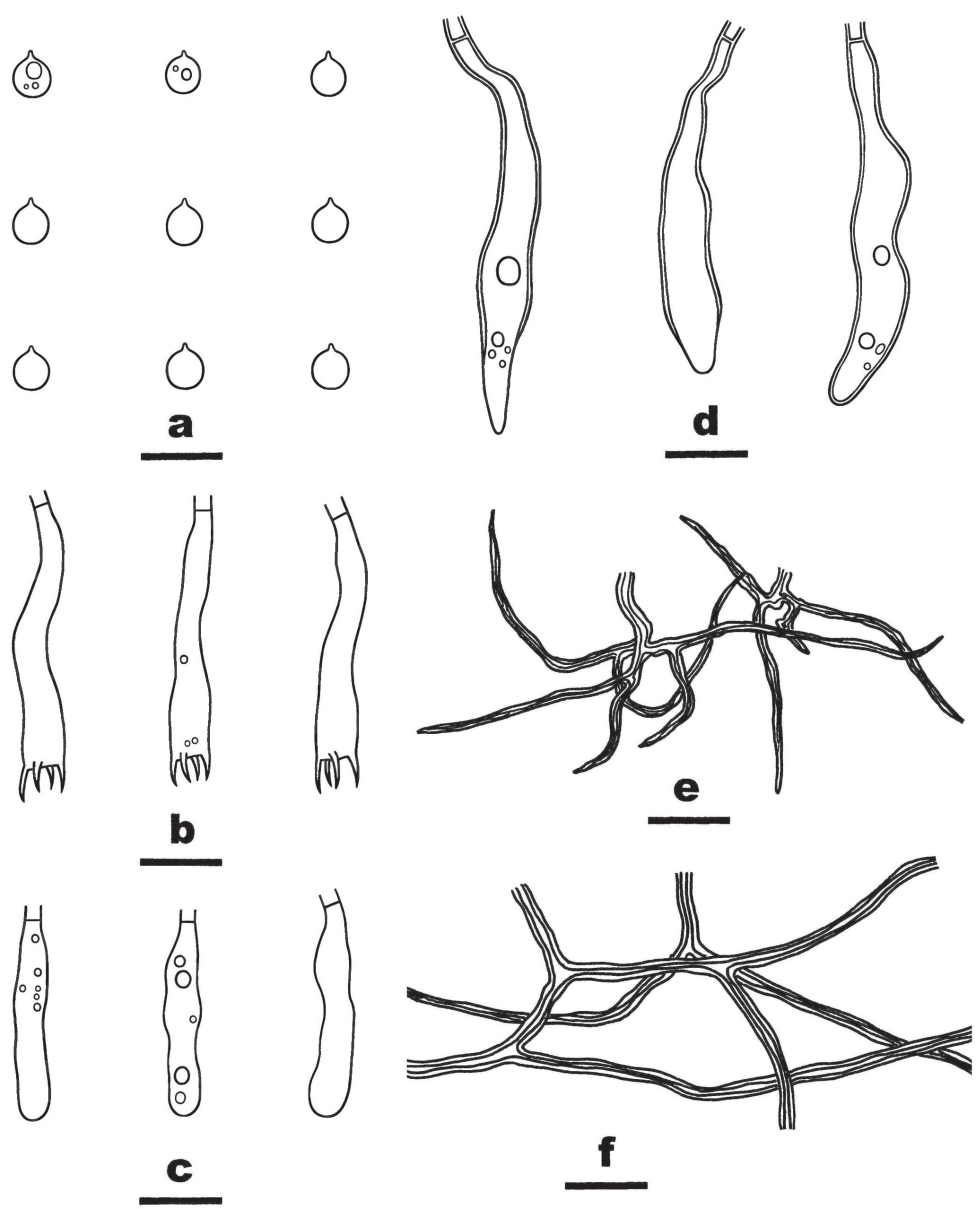
Microscopic structures of *Scytinostromaboidinii* (from the holotype He 6911) **a** basidiospores **b** basidia **c** basidioles **d** gloeocystidia **e** skeletal hyphae from hymenium **f** skeletal hyphae from subiculum. Scale bars: 10 µm (**a–f**).

##### Etymology.

Named to honor Dr. Jacques Boidin (Lyon, France) who contributed much to the taxonomy of *Scytinostroma*.

##### Fruiting body.

Basidiomes annual, resupinate, widely effused, closely adnate, inseparable from substrate, membranaceous to coriaceous, first as small patches, later confluent up to 9 cm long, 3.5 cm wide, up to 300 µm thick in section. Hymenophore smooth, pale yellow (4A3), greyish yellow (4B4) to greyish orange [5B(3–4)], unchanged in KOH, not cracked; margin thinning out, adnate, fimbriate, white or concolorous with hymenophore surface. Context pale yellow.

##### Microscopic structures.

Hyphal system dimitic. Context thickening, compact. Generative hyphae rare, scattered, simple-septate, colorless, slightly thick-walled, 2–3 µm in diam., IKI–, CB–. Skeletal hyphae dominant, colorless to yellow, distinctly thick-walled, moderately branched, 1.5–2 µm in diam., dextriniod, CB+. Catahymenium composed of skeletal hyphae, gloeocystidia, basidia and basidioles. Skeletal hyphae abundant, similar to those in the context, but strongly dextrinoid, dichotomous-branched with acute tips, 1–1.5 µm wide at lowest part. Gloeocystidia abundant, subcylindrical to subfusiform, colorless, slightly thick-walled, with or without contents, weakly SA+, 50–80 × 5–10 µm. Basidia subclavate to subcylindrical, thin-walled, colorless, smooth, with four sterigmata and a basal simple septum, 30–50 × 4–7 µm; basidioles in shape similar to basidia, but slightly smaller. Basidiospores subglobose, with a distinct apiculus, thin-walled, colorless, smooth, occasionally with oil-drops, amyloid, CB–, (4.5–) 5–5.5 (–6.5) × (4–) 4.5–5.5 (–6.2) µm, L = 5.1 µm, W = 5.0 µm, Q = 1.02–1.04 (n = 60/2).

##### Additional specimens examined.

China, Beijing, Mentougou District, Lingshan Scenic Spot, on dead angiosperm branch, 10 April 2022, He 7465a (BJFC 038600) & He 7465b (BJFC 038601); Gansu Province, Tianshui County, Dangchuan Forest Farm, on dead *Quercus* tree, 9 August 2015, He 2499 (BJFC 020952); Hebei Province, Xinglong County, Wulingshan Nature Reserve, on dead angiosperm branch, 2 September 2017, He 4985 (BJFC 024503); Jilin Province, Jiaohe County, forestry experimental area, on fallen angiosperm trunk, 3 September 2017, He 5138 (BJFC 024656).

##### Notes.

*Scytinostromaboidinii* is characterized by the relatively long gloeocystidia and subglobose basidiospores. In the phylogenetic tree (Fig. 1), *S.boidinii* formed a distinct lineage sister to *S.alutum* Lanq., which differs in having cracked basidiomes, slightly larger basidiospores (5–7 × 5–7.5 µm) and a distribution in France, Spain and Pakistan (Boidin and Lanquetin 1987; Bernicchia and Gorjón 2010). *Scytinostromayunnanense* C.L. Zhao from Yunnan Province, southwestern China, has similar-sized basidiospores (4.5–5.5 × 4.2–5.2 µm) to *S.boidinii*, but differs in having white to cream hymenophore, smaller gloeocystidia (28–33 × 4–5 µm) and smaller basidia (21–28 × 4–5.5 µm, Wang et al. 2020).

#### ﻿ Scytinostroma subduriusculum

Taxon classificationFungiRussulalesPeniophoraceae

Yue Li, S.L. Liu & S.H. He sp. nov.

D62AC5AE-22AB-5CAD-AA38-107223C3537E

 848266

Figs 6 , 7

##### Type.

China, Beijing, Haidian District, Beijing Botanical Garden, on dead angiosperm branch, 15 July 2022, He 7657 (BJFC 038793, holotype).

##### Etymology.

Refers to the morphological similarity and close phylogenetic relationship with *S.duriusculum*.

**Figure 6.**
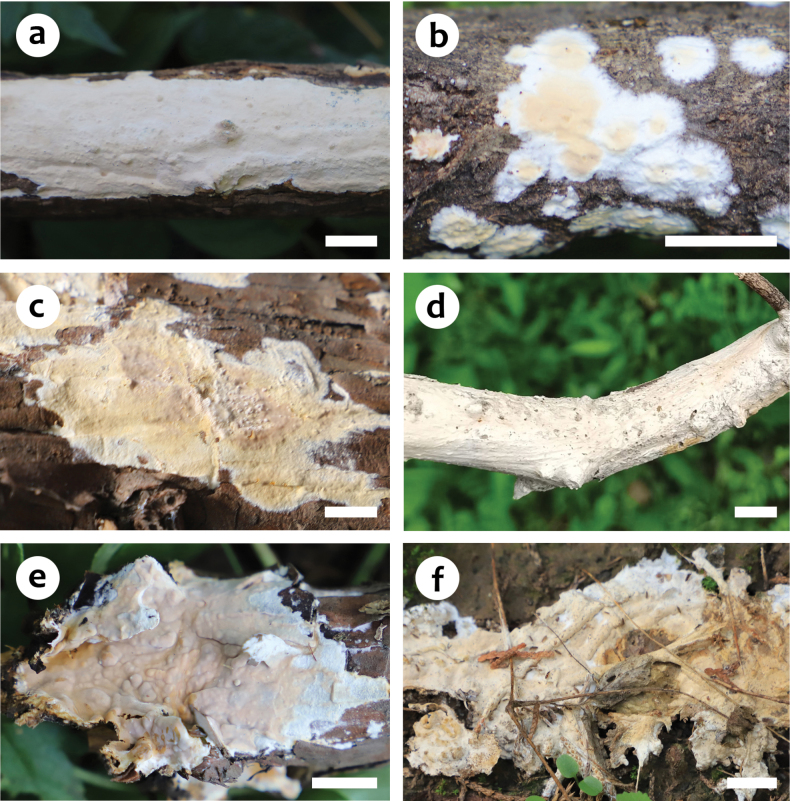
Basidiomes of *Scytinostromasubduriusculum***a** He 7657 (BJFC 038793, holotype) **b** He 7134 (BJFC 036451) **c** He 7141 (BJFC 036458) **d** He 7148 (BJFC 036465) **e** He 7150 (BJFC 036467) **f** He 7717 (BJFC 036467). Scale bars: 1 cm (**a–f**).

**Figure 7.**
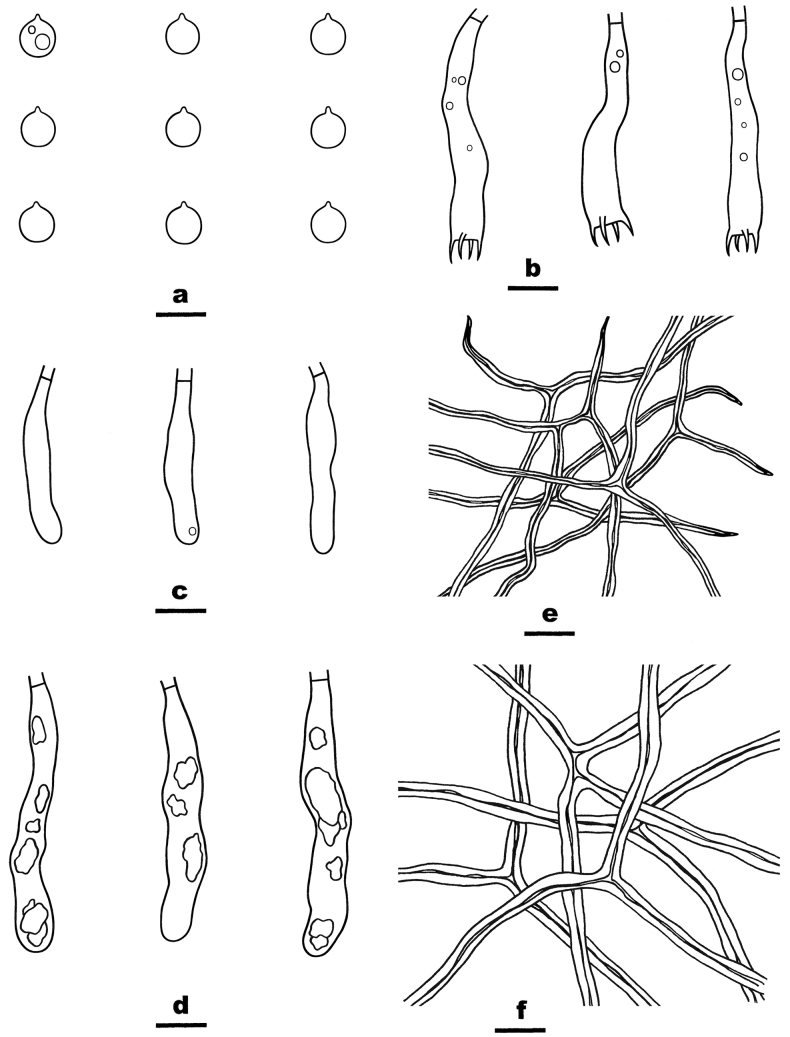
Microscopic structures of *Scytinostromasubduriusculum* (from the holotype He 7657) **a** basidiospores **b** basidia **c** basidioles **d** gloeocystidia **e** skeletal hyphae from hymenium **f** skeletal hyphae from subiculum. Scale bars: 10 µm (**a–f**).

##### Fruiting body.

Basidiomes annual, resupinate, widely effused, closely adnate, inseparable from substrate, membranaceous to coriaceous, first as small patches, later confluent up to 18 cm long, 3 cm wide, up to 160 µm thick in section. Hymenophore smooth, light yellow (4A4) to greyish orange (5B4), unchanged in KOH, not cracked; margin thinning out, adnate, fimbriate, white or concolorous with hymenophore surface. Context pale yellow.

##### Microscopic structures.

Hyphal system dimitic. Context thickening, compact. Generative hyphae rare, scattered, simple-septate, colorless, thin-walled, 2–3 µm in diam., IKI–, CB–. Skeletal hyphae dominant, colorless to pale yellow, distinctly thick-walled, moderately branched, 2.5–4.5 µm in diam., dextriniod, CB+. Catahymenium composed of skeletal hyphae, gloeocystidia, basidia and basidioles. Skeletal hyphae abundant, similar to those in the context, but strongly dextrinoid, 1–1.5 µm in diam. Gloeocystidia abundant, subclavate to subcylindrical, colorless, thin-walled, usually with contents, SA+, 50–70 × 6–9 µm. Basidia subclavate to subcylindrical, slightly curved, thin-walled, colorless, smooth, with four sterigmata and a basal simple septum, 30–45 × 6–7.5 µm; basidioles in shape similar to basidia, but slightly smaller. Basidiospores subglobose, with a distinct apiculus, thin-walled, colorless, smooth, occasionally with oil-drops, amyloid, CB–, (6–) 6.2–7 (–7.5) × (5.5–) 5.8–6.8 (–7) µm, L = 6.5 µm, W = 6.3 µm, Q = 1.01–1.04 (n = 90/3).

##### Additional specimens examined.

CHINA, Beijing, Changping District, Baiyanggou Scenic Spot, on dead angiosperm branch, 21 July 2021, He 7134 (BJFC 036451); Daxing District, Nanhaizi Park, on dead angiosperm branch, 31 July 2021, He 7148 (BJFC 036465); Fangshan District, Qinglonghu Park, on dead Sabina branch, 31 July 2021, He 7150 (BJFC 036467); Fengtai District, Yungang Forest Park, on dead *Sabina* tree, 25 July 2021, He 7141 (BJFC 036458); Haidian District, Bajia Country Park, on *Sabina* stump, 16 August 2022, He 7717 (BJFC 038853); Guangxi Autonomous Region, on dead angiosperm branch, 16 June 2016, He 3819 (BJFC022318); Guizhou Province, Libo Country, Xiaoqikong Scenic Spot, on dead angiosperm branch, 16 June 2016, He 3822 (BJFC022321); Hainan Province, Baoting Country, Qixianling Forest Park, on dead angiosperm branch, 18 March 2016, He 3590 (BJFC022090) & He 3593 (BJFC022092); Haikou City, Jinniuling Park, on dead twig of living *Araucaria*, 7 June 2016, He 3825 (BJFC022327); Wanning City, Xinglong Tropical Botanical Garden, on dead angiosperm branch, 19 March 2016, He 3603 (BJFC022101); Yunnan Province, Qiubei Country, Puzhehei Scenic Spot, on dead angiosperm branch, 26 July 2014, He 20140726-5 (BJFC019218) & He 20140726-6 (BJFC019219); Ruili City, Moli Tropical Rain Forest Scenic Spot, on fallen angiosperm trunk, 2 December 2015, He 3497 (BJFC021894). THAILAND, Chiang Rai, Mae Fah Luang University, on fallen angiosperm trunk, 21 July 2016, He 4045 (BJFC023484); Krabi, on dead angiosperm branch, 28 July 2016, He 4146 (BJFC023588). VIETNAM, Ho Chi Minh City Animal and Botanical Garden, on fallen angiosperm trunk, 4 September 2017, He 5204 (BJFC024722).

##### Notes.

*Scytinostromasubduriusculum* is characterized by subcylindrical gloeocystidia, subglobose, relatively large basidiospores, and growth on both angiosperm and gymnosperm trees. It is widely distributed in China, and also found in Thailand and Vietnam. In the phylogenetic tree (Fig. 1), *S.subduriusculum* formed a distinct lineage sister to *S.duriusculum* (Berk. & Broome) Donk. There are 24 base pair differences between *S.subduriusculum* (He 4146, Thailand) and *S.duriusculum* (He 5748, Sri Lanka), and 66 differences between *S.subduriusculum* and *S.duriusculum* (CBS 757.81, France) of total 665 base pairs of ITS1+5.8S+ITS2 sequences. The similarities are 96.4% (He 4146 vs. He 5748) and 90.1% (He 4146 vs. CBS 757.81). The French samples may represent an undescribed species because their ITS1+5.8S+ITS2 sequences largely differ from those of Sri Lankan samples (71 differences of total 665 base pairs with only 89.3% similarity). Morphologically, the two species share subclavate to subcylindrical gloeocystidia and subglobose basidiospores. However, *S.duriusculum* has shorter basidia (20–25 µm) and smaller basidiospores (6–6.5 × 5.5–6 µm, measured from He 5748 from Sri Lanka).

#### ﻿ Scytinostroma subrenisporum

Taxon classificationFungiRussulalesPeniophoraceae

Yue Li, S.L. Liu & S.H. He sp. nov.

81546DD1-EFA3-5DDA-B160-92CA0A65D4A7

 848269

Fig. 8

##### Type.

China, Guizhou Province, Libo County, Maolan Nature Reserve, on dead angiosperm branch, 11 July 2017, He 4792 (BJFC 024311, holotype).

**Figure 8.**
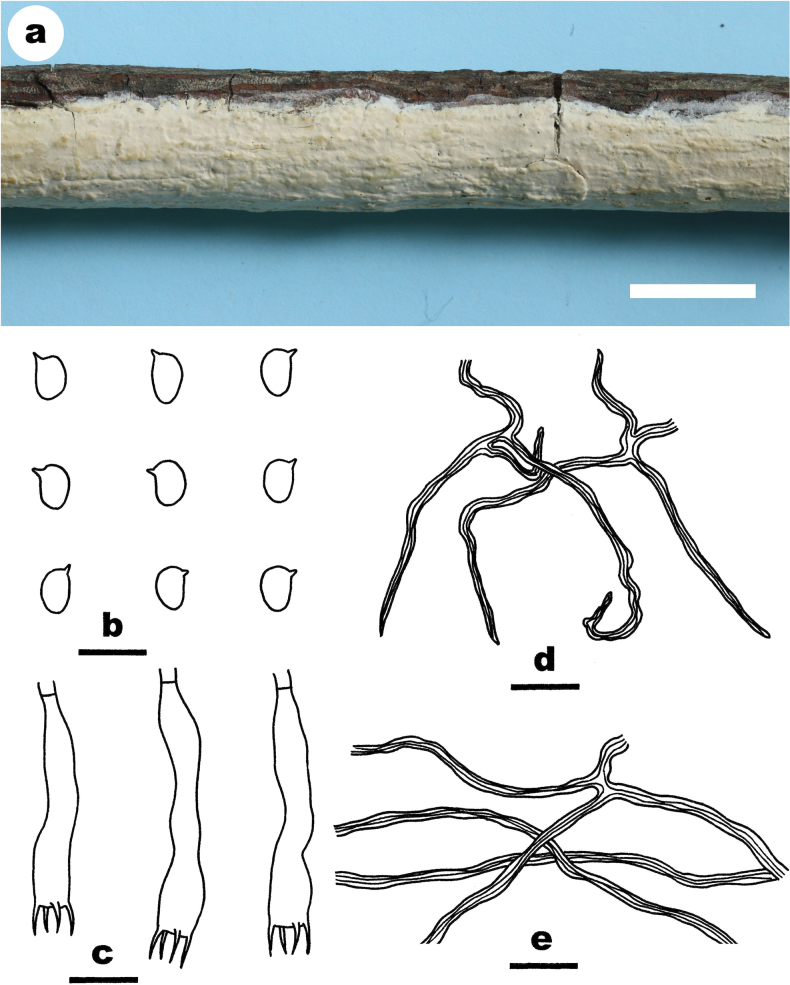
*Scytinostromasubrenisporum* (from the holotype He 4792) **a** basidiomes **b** basidiospores **c** basidia **d** skeletal hyphae from hymenium **e** skeletal hyphae from subiculum. Scale bars: 1 cm (**a**); 10 µm (**b–e**).

##### Etymology.

Refers to the morphological similarity and close phylogenetic relationship with *S.renisporum*.

##### Fruiting body.

Basidiomes annual, resupinate, widely effused, closely adnate, inseparable from substrate, membranaceous to coriaceous, first as small patches, later confluent up to 10 cm long, 2.5 cm wide, up to 100 µm thick in section. Hymenophore smooth, pale orange (5A3), light orange (5A4) to greyish orange [5B(5–6)], unchanged in KOH, not cracked; margin thinning out, adnate, fimbriate, white or concolorous with hymenophore surface. Context pale yellow.

##### Microscopic structures.

Hyphal system dimitic. Context thickening, compact. Generative hyphae rare, scattered, simple-septate, colorless, thin-walled, 2–3 µm in diam., IKI–, CB–. Skeletal hyphae dominant, colorless to yellow, distinctly thick-walled, moderately branched, 1.5–2 µm in diam., dextriniod, CB+. Catahymenium composed of skeletal hyphae, basidia and basidioles. Skeletal hyphae abundant, similar to those in the context, but strongly dextrinoid, moderately branched with acute tips, 1.5–2 µm wide at lowest part. Gloeocystidia absent. Basidia subcylindrical, slightly curved, thin-walled, colorless, smooth, with four sterigmata and a basal simple septum, 35–45 × 4.5–6.5 µm; basidioles in shape similar to basidia, but slightly smaller. Basidiospores ellipsoid to reniform, with a distinct apiculus, thin-walled, colorless, smooth, amyloid, CB–, (5.5–) 6–6.5 (–7) × (3.8–) 4–5 (–5.5) µm, L = 6.2 µm, W = 4.4 µm, Q = 1.35–1.45 (n = 60/2).

##### Additional specimens examined.

China, Anhui Province, Qimen County, Guniujiang Nature Reserve, on dead angiosperm branch, 8 August 2013, He 1720 (BJFC 016187); Fujian Province, Wuyishan County, Wuyishan Nature Reserve, on dead angiosperm branch, 3 October 2018, He 5685 (BJFC 026747) & He 5686 (BJFC 026748); Guangxi Autonomous Region, Huanjiang County, Mulun National Nature Reserve, on dead angiosperm branch, 10 July 2017, He 4751 (BJFC 024270); Guizhou Province, Libo County, Maolan Nature Reserve, on angiosperm tree, 15 June 2016, He 3792 (BJFC 022291); Hunan Province, Guzhang County, Gaowangjie National Nature Reserve, on dead angiosperm branch, 3 August 2018, He 5626 (BJFC 026688); Jiangxi Province, Ji’an County, Jinggangshan Scenic Spot, on dead angiosperm branch, 11 August 2016, He 4303 (BJFC 023745); Lianping County, Jiulianshan Nature Reserve, on dead *Liana* branch, 14 August 2016, He 4384 (BJFC 023825); Yifeng County, Guanshan Nature Reserve, on dead angiosperm branch, 9 August 2016, He 4170 (BJFC 023612) & He 4199 (BJFC 023641).

##### Notes.

*Scytinostromasubrenisporum* is characterized by the absence of gloeocystidia and ellipsoid to reniform basidiospores. In the phylogenetic tree (Fig. 1), *S.subrenisporum* is closely related to *S.acystidiatum* and *S.renisporum*. *Scytinostromaacystidiatum* that was recently described from China, is similar to *S.subrenisporum* by sharing the absence of gloeocystidia but differs in having smaller basidiospores (5.2–6 × 3.5–4.5 µm, measured from the type by the authors, Zhang et al. 2023). *Scytinostromarenisporum* is similar to *S.subrenisporum* by sharing ellipsoid to reniform basidiospores but differs by having cylindrical, subclavate or fusoid gloeocystidia and a distribution in Côte d’Ivoire, western Africa (Boidin and Lanquetin 1987).

## ﻿Discussion

Previous studies showed that *Scytinostroma* is polyphyletic (Larsson and Larsson 2003). In this study, we performed phylogenetic analyses of Peniophoraceae based on ITS1-5.8S-ITS2-nrLSU sequences of samples of *Scytinostroma* s.s. and representative taxa of other genera. Species of *Scytinostroma* s.s. including the type, *S.portentosum*, formed a moderately supported clade in the likelihood analysis but a strongly supported clade in the Bayesian analysis. For the moment, we prefer to treat this clade as a monophyletic genus and believe that the support values could be higher if more samples of Peniophoraceae are included. Since a sequence of *S.portentosum* (type species, described from Pennsylvania) from Canada was distinct from the strongly supported sister lineage comprised of sequences of *S.portentosum* and *S.hemidichophyticum* from Europe, we suppose that all the European sequences represent *S.hemidichophyticum* and *S.portentosum* is restricted in distribution to North America. The two species, *S.artocreas* and *S.incrustatum* transferred from *Michenera* by Stalpers et al. (2021) based on morphological evidence, were nested within the *Scytinostroma* s.s. *Scytinostromaduriusculum* is a cosmopolitan species and has been reported from many countries in subtropical and tropical areas (Boidin and Lanquetin 1987; Bernicchia and Gorjón 2010). However, our phylogenetic analyses demonstrated that it could be a species complex, because three lineages were recognized from the samples of France, Sri Lanka, and China, Thailand and Vietnam.

On the one hand, species in *Scytinostroma* s.s. clade have some common morphological characters, for example, simple-septate generative hyphae, and ovoid, reniform to subglobose basidiospores with amyloid reactions in Melzer’s reagent. However, this doesn’t mean species with inamyloid basidiospores could not belong to *Scytinostroma* s.s. On the other hand, the shape of skeletal hyphae, presence of gloeocystidia and encrusted cystidia, and size of basidiospores varies in different species. Based on our phylogenetic and morphological study results, we recognized 14 species of *Scytinostroma* s.s.worldwide. Until now, seven species have been reported from China, all of which were newly described in the present study and other recently published papers (Liu et al. 2018; Wang et al. 2020; Zhang et al. 2023). It seems that China, especially its temperate areas, is rich in species diversity of *Scytinostroma* s.s. Although *Scytinostroma* s.s. is well studied in the present paper, the species diversity, taxonomy and phylogeny of *Scytinostroma* s.l. and related genera are still unresolved. A comprehensive study on this issue is urgently needed.

### ﻿A key to species of *Scytinostroma* s.s. worldwide

**Table:** 

1	Gloeocystidia absent	**2**
–	Gloeocystidia present	**3**
2	Basidiospores > 6 µm long	***S.subrenisporum***
–	Basidiospores < 6 µm long	***S.acystidiatum***
3	Basidiospores > 15 µm long	**4**
–	Basidiospores < 15 µm long	**6**
4	Basidiospores fusiform to navicular	***S.caudisporum***
–	Basidiospores subglobose to globose	**5**
5	Lamprocystidia present, basidiospores 17–22 × 16–21 µm	***S.incrustatum***
–	Lamprocystidia absent, basidiospores 16–19 × 14–16 µm	***S.artocreas***
6	Basidiospores ovoid to reniform	***S.renisporum***
–	Basidiospores subglobose	**7**
7	Distributed in subtropical and tropical areas	**8**
–	Distributed in temperate areas	**10**
8	Gloeocystidia < 50 µm long	***S.yunnanense***
–	Gloeocystidia > 50 µm long	**9**
9	Reported from Sri Lanka, basidia 20–25 µm long, basidiospores 6–6.5 × 5.5–6 µm	***S.duriusculum***
–	Reported from China, Thailand, Vietnam, basidia 30–45 µm long, basidiospores 6.2–7 × 5.8–6.8 µm	***S.subduriusculum***
10	Reported from occidental countries	**11**
–	Reported from China	**13**
11	North American species	***S.portentosum***
–	European species	**12**
12	Skeletal hyphae in hymenium rarely branched	***S.alutum***
–	Skeletal hyphae in hymenium dichotomously branched	***S.hemidichophyticum***
13	Gloeocystidia two kinds	***S.beijingensis***
–	Gloeocystidia one kind	**14**
14	Basidiospores > 6 µm in diam.	***S.subduriusculum***
–	Basidiospores < 6 µm in diam.	***S.boidinii***

## Supplementary Material

XML Treatment for Scytinostroma beijingensis

XML Treatment for Scytinostroma boidinii

XML Treatment for Scytinostroma subduriusculum

XML Treatment for Scytinostroma subrenisporum

## ﻿Additional information

### Author ORCIDs

Yue Li https://orcid.org/0000-0003-4091-1506

Wei-Qi Xu https://orcid.org/0009-0000-5675-386X

Shi-Liang Liu https://orcid.org/0000-0001-7556-2575

Ning Yang https://orcid.org/0009-0007-6900-5669

Shuang-Hui He https://orcid.org/0000-0003-4702-3034

### Data availability

All of the data that support the findings of this study are available in the main text or Supplementary Information.
